# Organoid-based epithelial to mesenchymal transition (OEMT) model: from an intestinal fibrosis perspective

**DOI:** 10.1038/s41598-017-02190-5

**Published:** 2017-05-26

**Authors:** Soojung Hahn, Myeong-Ok Nam, Jung Hyun Noh, Dong Hyeon Lee, Hyun Wook Han, Duk Hwan Kim, Ki Baik Hahm, Sung Pyo Hong, Jun-Hwan Yoo, Jongman Yoo

**Affiliations:** 1 Department of Microbiology, School of Medicine, CHA University, Seongnam, South Korea; 2 Institute of Basic Medical Sciences, School of Medicine, CHA University, Seongnam, South Korea; 3 Department of Physiology, School of Medicine, CHA University, Seongnam, South Korea; 4 Department of Preventive Medicine, School of Medicine, CHA University, Seongnam, South Korea; 5 Department of Gastroenterology, CHA Bundang Medical Center, CHA University, Seongnam, South Korea

## Abstract

The current *in vitro* or *in vivo* intestinal fibrosis models have many limitations. Recent advancements in the isolation and culturing of organoids has led to development of various three-dimensional (3D) intestinal disease models with *in vivo* physiology. In this study, we generated an organoid-based epithelial to mesenchymal transition (OEMT) model, which could be used as a novel intestinal fibrosis model. Intestinal epithelial organoids (IEOs) were isolated and cultured from the small intestines of normal mice. IEOs were treated with transforming growth factor- β1 (TGF-β1) or Tumor necrosis factor-α (TNF-α) to evaluate their phenotypic change. Raw 264.7 cells (macrophage) stimulated with lipopolysaccharide were co-cultured with IEOs in growth media with or without TGF-β1. TGF-β1 alone slightly induced epithelial to mesenchymal transition (EMT) in the IEOs but mainly disrupted them. Macrophage released cytokines synergistically induced mesenchymal phenotypic changes in TGF-β1 stimulated intestinal organoids. TNF-α and TGF-β1 synergistically induced proliferation of mesenchymal cells as well as EMT in the IEOs. We generated a novel OEMT model based on our finding that TNF-α and TGF-β synergistically induce type 2 EMT in IEOs. This 3D EMT model with *in vivo* physiology could be used to study EMT associated intestinal fibrosis.

## Introduction

Intestinal fibrosis is a common complication of inflammatory bowel diseases (IBDs) such as in Crohn’s disease (CD) and ulcerative colitis (UC)^1^. Although intestinal fibrosis is one of the major causes decreasing the quality of life as well as increasing the morbidity and mortality of IBD patients, there is no FDA approved anti-fibrotic agent that can prevent or treat intestinal fibrosis^1–3^. To develop anti-fibrotic agents, appropriate experimental models for intestinal fibrosis are needed to confirm their efficacy; however, the fibrosis models currently used have many limitations for use in the screening of anti-fibrotic agents. The most widely used cellular models of intestinal fibrosis activate the fibrogenic responses of myofibroblasts by treating them with transforming growth factor-β1 (TGF-β1) or mechanical stress^4–6^. However, these models do not fully recapitulate *in vivo* physiology because they do not reflect the three-dimensional (3D) intestinal structure including multiple, specialized cell types. Even *in vivo* models of intestinal fibrosis, including the commonly used trinitrobenzene sulfonic acid (TNBS)^7–9^ and Salmonella typhimurium models^9,10^, are not practical for large-scale drug screening because they do not represent human pathophysiology and are inefficient and expensive^4^.

Recent advances in long-term 3D culture of isolated intestinal crypts or intestinal stem cells have allowed the generation of human intestinal epithelial organoids (IEOs). This 3D culture model recapitulates human *in vivo* physiology because the IEOs contain all the intestinal epithelial cell types differentiated and proliferated from intestinal stem cells^11,12^. Thus far, this model has been extensively used for generating intestinal epithelial disease models as well as studying intestinal stem cell self-renewal, growth and differentiation^13^. However, little has been done on developing a suitable intestinal fibrosis model based on IEOs. Recently, Eva S. *et al*. reported on a novel model of intestinal fibrosis using IEOs differentiated from pluripotent embryonic stem cells. The model contains myofibroblasts as well as diverse epithelial cells but still requires high technology for the handling of pluripotent stem cells and many complex experimental steps as well as has a long duration and high costs^4,14,15^.

Type 2 epithelial to mesenchymal transition (EMT) is known as one of the key mechanisms of intestinal fibrosis. Activated myofibroblasts, which are key effector cells in intestinal fibrosis and produce excessive amounts of collagen-rich extracellular matrix, can be derived from epithelial cells by EMT. During EMT, epithelial cells gain a spindle-shape morphologic change and mesenchymal cell markers such as vimentin and α-smooth muscle actin (α-SMA) and lose epithelial cell markers^16,17^. Although EMT is one of main mechanisms of intestinal fibrosis, previous *in vitro* models based on EMT-associated intestinal fibrosis have limited utility because they use immortalized epithelial cell lines^6,18,19^ due to the rapid death of isolated primary epithelial cells in *in vitro* cultures^20^. Immortalization often leads to changes in gene expression profiles and altered responses; thus, the cell lines do not represent primary cells^13^.

In this study, we generated an organoid-based epithelial to mesenchymal transition (OEMT) model, which can be used to study intestinal fibrosis, using tumor necrosis factor-α (TNF-α) and TGF-β. This model may represent a bridge model to develop a cost-efficient alternative tool with human *in vivo* physiology for the screening of anti-fibrotic agents.

## Results

### TGF-β1 treatment induced the disruption of IEOs in a concentration- and time-dependent manner

**Figure 1:**
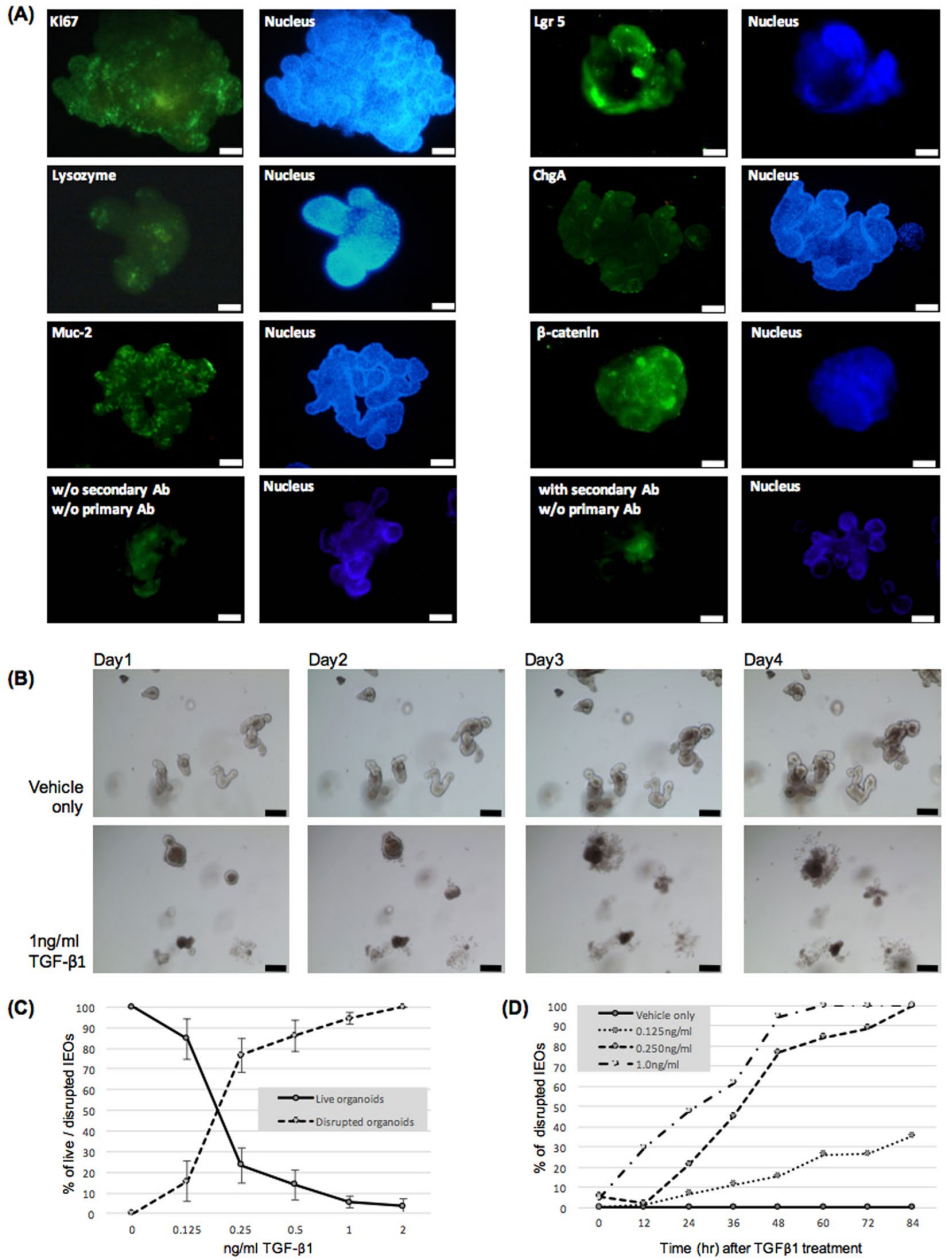
Disruption of IEOs by TGF-β1 treatment. (**A**) Cellular components of IEOs were stained with various cell markers constituting the intestinal epithelium. Bars, 50 μm. (**B**) Representative images showing IEOs treated with or without TGF-β. Bars, 100 μm. (**C**) Concentration-dependency of the TGF-β1-mediated IEO disruption. The number of live and disrupted organoids was counted by concentration after 48 hours TGF-β1 treatment. (**D**) Time-dependency of the TGF-β1-mediated IEO disruption. The number of live and disrupted organoids was counted by time after the treatment. Data are presented as the mean ± SEM; n = 3 samples per bar.

Because TGF-β1 induced pathological EMT in epithelial cell lines in previous cancer cell line experiments^21^, we examined whether TGF-β1 could also mediate mesenchymal transition in IEOs. To explore the TGF-β1 mediated EMT of IEOs, we cultured IEOs originating from the isolated crypts of mouse small intestines. IEOs were immunostained for Ki67, Lgr5, muc-2, lysozyme, chromogranin A (ChgA), and β-catenin as markers for proliferating cells, intestinal stem cells, goblet cells, paneth cells, enteroendocrine cells, and epithelial cells, respectively^22,23^. The immunostaining results show that all these cells were present in the IEOs (Fig. 1A).

To examine the effects of TGF-β1 in the IEOs, we added 1 ng/ml of TGF-β1 to the IEO growth medium 2 days after the initial seeding of the IEOs and temporally tracked the morphologic changes. We observed that the number of disrupted or dead organoids were higher in the TGF-β1 treated IEOs than in the vehicle-only (DPBS) treated control IEOs (Fig. 1B). The concentration-dependency of the TGF-β1-mediated IEO disruption was determined with TGF-β1 concentrations of 0, 0.125, 0.25, 0.5, 1.0 and 2.0 ng/ml, and live and disrupted IEOs were quantified at 12 h intervals post treatment. As shown in Fig. 1C and D, TGF-β1 induced the disruption of IEOs in a concentration-dependent manner. Our results show that the effects of the TGF-β1 treatment mainly were the disruption and cell death of IEOs in contrast to the previously reported results from cancer cell lines (21).

### Macrophage-released cytokines can synergistically induce mesenchymal phenotypic changes in TGF-β1 treated IEOs

**Figure 2:**
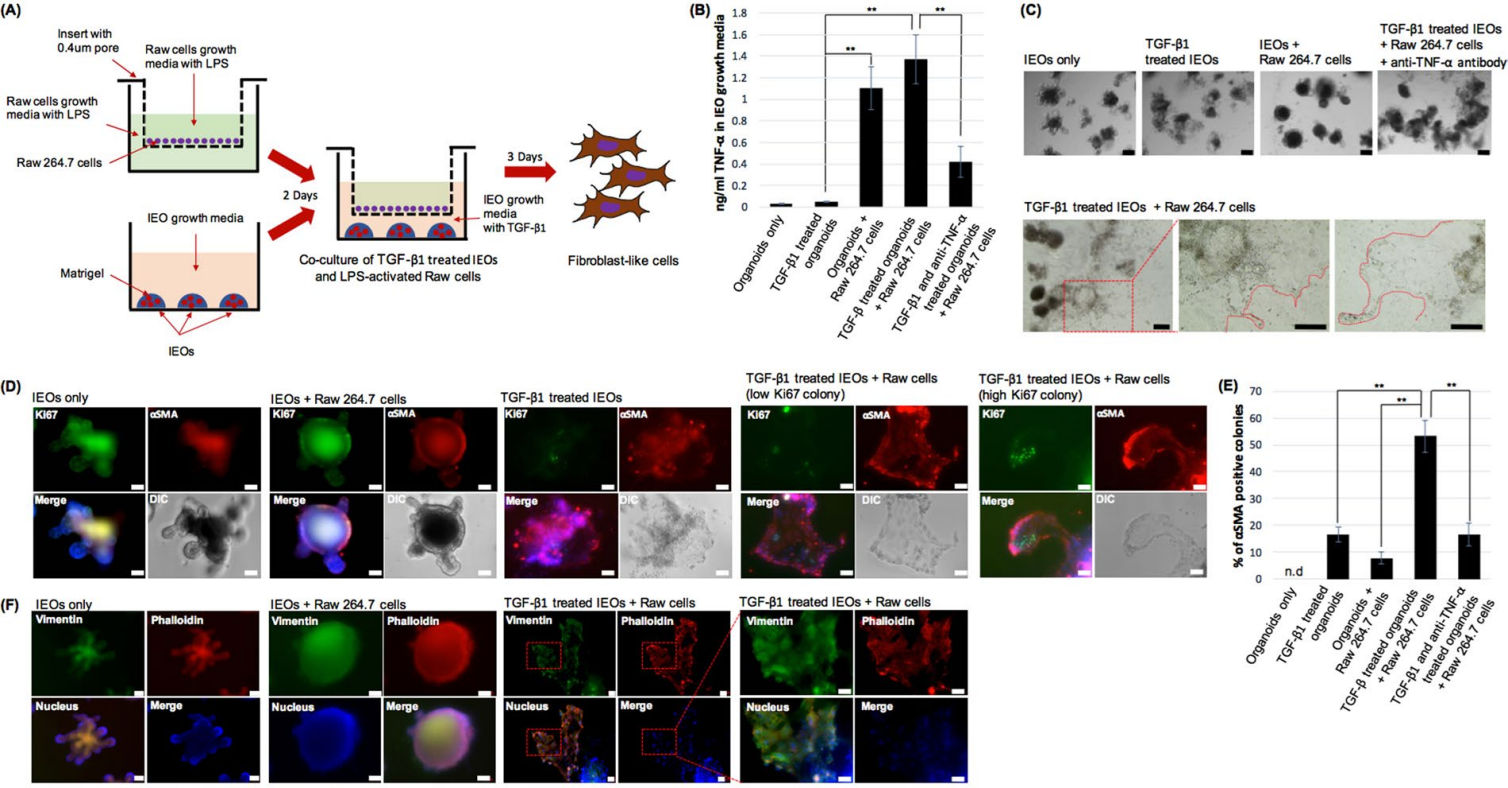
Macrophage released cytokines can synergistically induce mesenchymal phenotypic changes in TGF-β treated IEOs. (**A**) A schematic illustration of the OEMT model with IEOs co-cultured with RAW264.7 cells. (**B**) Quantitation of TNF-α in the culture medium from TGF-β treated IEOs with or without RAW264.7 cells or anti-TNF-α neutralizing antibody. (**C**) Representative images showing the IEOs following TGF-β treatment and RAW 264.7 cells. Bars, 100 μm. (**D**) The expression of α-SMA and Ki67 was examined in TGF-β1 treated IEOs with RAW 264.7 cells. Bars, 50 μm. (**E**) The percentage of α-SMA positive colonies was calculated. Data are presented as the mean ± SEM; n = 4 samples per bar, **p < 0.01, *p < 0.05. (**F**) The expression of vimentin and F-actin reorganization was examined in TGF-β1 treated IEOs with RAW 264.7 cells. Bars, 50 μm.

Previous studies have suggested that the inflammatory microenvironment and TGF-β1 cooperatively induce pathological EMT^24^. Based on these results, we speculated that pro-inflammatory cytokines would provide great inducers in TGF-β1 treated IEOs. To test this hypothesis, we established a three-dimensional co-culture system in which TGF-β1 treated IEOs derived from mouse small intestines were maintained in a Matrigel matrix, and LPS-stimulated RAW 264.7 cells, a murine macrophage cell line, was grown on a 0.4 um pore size transwell (Fig. 2A). The IEO growth medium co-cultured with RAW 264.7 cells had a significantly higher level of TNF-α, a proinflammatory cytokine, than that of the IEO growth medium without RAW 264.7 cells at 1 day after the co-cultivation. It was significantly lowered by adding TNF-α neutralizing antibody (Fig. 2B). We first examined morphologically whether the RAW 264.7 cell secreted cytokines increased the induction of mesenchymal changes. Mesenchymal-like cells in TGF-β1 treated IEOs with RAW 264.7 cells were observed more frequently than those in TGF-β1 treated IEOs without RAW 264.7 cells. However, the number of mesenchymal-like cells in IEOs treated only with TGF-β1 is almost the same as those treated with the combination of TGF-β1, TNF-α neutralizing antibody, and RAW 264.7 cells (Fig. 2C).

When IEOs and IEO derived colonies were immunostained for both α-SMA, a marker for mesenchymal cells, and Ki67, a marker for proliferating cells, α-SMA expression in nearly all the mesenchymal like colonies was observed in the TGF-β1 treated IEOs with RAW264.7 cells. However, we also observed that the Ki67 expressed colonies in the α-SMA expressed colonies were lower by about 30% compared to the vehicle-only treated IEOs which was almost 100% (Fig. 2D). α-SMA positive colony should meet the following criteria; 1) the IEOs morphology disappears, 2) the EMT-like morphology is visible, and 3) the fluorescence for α-SMA staining is visible. The α-SMA positive colonies were significantly higher in the TGF-β1 treated IEOs with Raw 264.7 cells than in the TGF-β1 treated IEOs without RAW264.7 cells (3.2-fold, n = 4, p < 0.01). However, when the TNF-α neutralizing antibody was added in TGF-β1 treated IEOs with RAW 264.7 cells, the number of α-SMA positive colonies was reduced to a similar number of α-SMA in TGF-β1 treated IEOs without RAW 264.7 cells (0.31-fold, n = 4, Fig. 2E). Lastly, when the IEOs and IEO derived colonies were immunostained for vimentin and filamentous actin (F-actin) fibers, another marker for mesenchymal cells, a similar result was also observed in the TGF-β1 treated IEOs with RAW264.7 cells (Fig. 2F). Taken together, these analyses suggest that TGF-β1 treated IEOs with RAW264.7 cells increased the mesenchymal changes compared with the TGF-β1 treated IEOs without any treatment. Moreover, TNF-α is an essential cytokine which increases TGF-β1 mediated mesenchymal changes by co-cultivating RAW264.7 cells.

### Pretreatment of TNF-α can synergistically induce mesenchymal phenotypic changes in TGF-β1 treated IEOs

**Figure 3:**
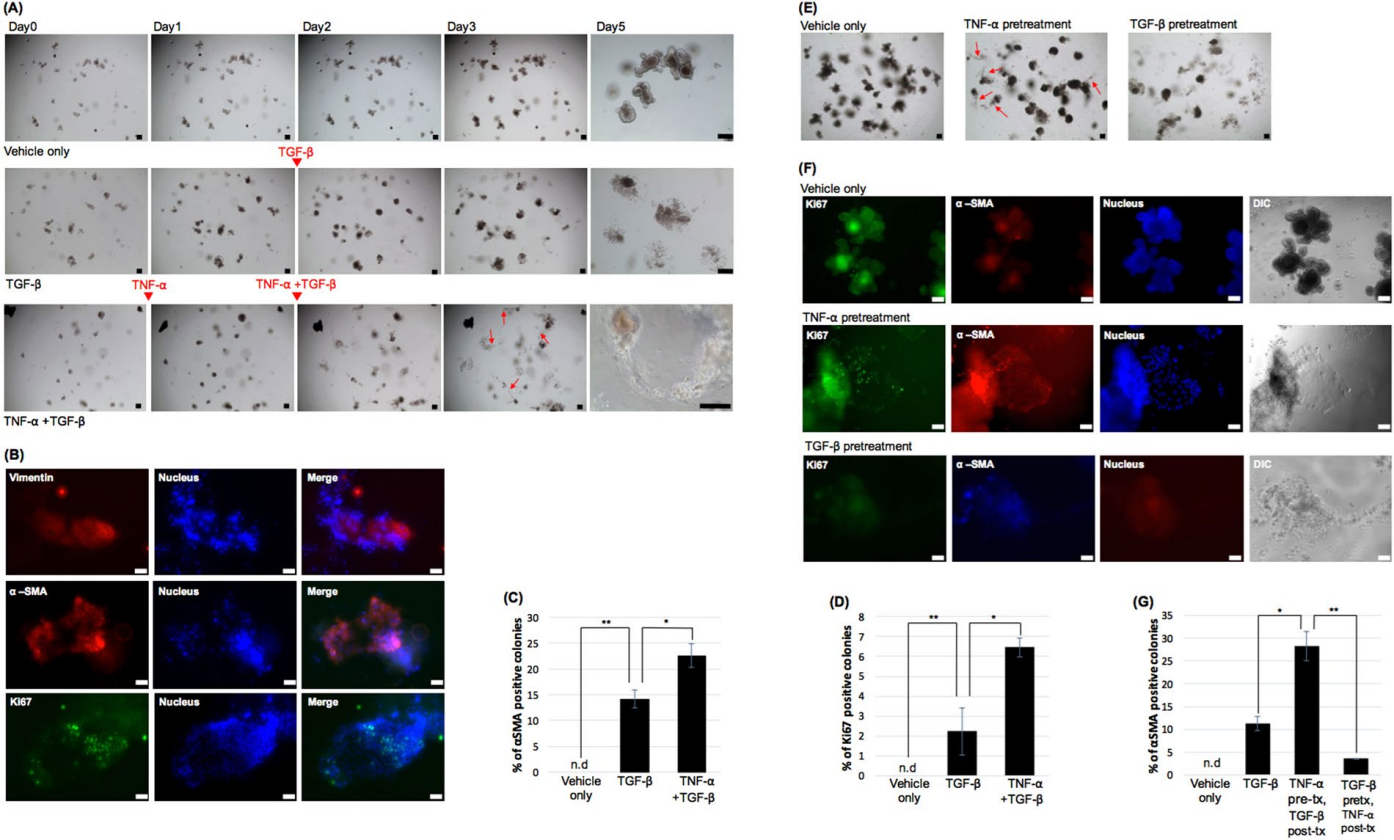
Pretreatment of TNF-α can synergistically induce mesenchymal changes in TGF-β treated IEOs. (**A**) Time-course images of the mesenchymal changes and migratory cells in IEOs treated with TNF-**α** and TGF-β1. Bars, 100 μm. Red arrow, migrating cells. (**B**) The expression of α-SMA and Ki67 was examined in TGF-β and TNF-α treated IEOs. Bars, 50 μm. (**C**) The percentage of α-SMA positive colonies among all the colonies was determined. (**D**) The percentage of Ki67 positive colonies among all the colonies was determined. (**E**) Images of mesenchymal change when the order of treatment of TNF-α and TGF-β in IEOs was changed. Bars, 100 μm. Red arrow, migrating cells. (**F**) The expression of α-SMA and Ki67 was examined when the order of treatment of TNF-α and TGF-β in IEOs was changed. Bars, 50 μm. (**G**) The percentage of α-SMA positive colonies among all the colonies was determined when the order of treatment of TNF-α and TGF-β in IEOs was changed. Data are presented as the mean ± SEM; n = 4 samples per bar, **p < 0.01, *p < 0.05.

A previous study on colon carcinoma spheroids derived from LIM1863 cells suggested that TNF-a stimulates TGF-β1-induced EMT and chemotactic migration^25^. Based on this result, we speculated that pretreatment with TNF-α before the TGF-β1 treatment in the IEOs would result in a greater induction of mesenchymal changes than that of the no-pretreatment. To confirm this, we examined whether mesenchymal changes and migration were observed when IEOs were treated with TNF-α and TGF-β1, respectively, on days 1 and 2 after the initial seeding of the IEOs. The number of mesenchymal changes and migratory cells in the IEOs treated with both TNF-α and TGF-β1 was greater than that of the TGF-β1 treated IEOs without TNF-α (Fig. 3A). When the TNF-α and TGF-β1 treated IEOs were immunostained for Vimentin, a-SMA and Ki67, almost all the mesenchymal changed colonies exhibited the mesenchymal and proliferation markers (Fig. 3B). An approximately 1.57-fold increase in the α-SMA + colonies was observed in the TNF-α and TGF-β1 treated group relative to the TGF-β1 treated group (n = 4, p < 0.05) (Fig. 3C), and a 2.89-fold increase in the Ki67 positive colonies was observed in the TNF-α and TGF-β1 treated group relative to the TGF-β1 treated group (n = 4, p < 0.05) (Fig. 3D).

Next, in order to examine whether the sequence of TNF-α and TGF-β1 treatment plays an important role in the EMT efficiency, we began with TNF-α followed by TGF-β1 on day one, and reversed the sequence one day later. No mesenchymal changes and migratory cells was observed when TGF-β1 was treated first and then treated with TNF-α. The similar results were shown in immunostaining with α-SMA and also in quantification of α-SMA positive colonies. This result suggests that the TNF-α pretreatment was effective in inducing mesenchymal changes in the TGF-β1 treated IEOs. Also, the result shows that the order of TNF-α and TGF-β1 treatment is critical.

### Pretreatment of TNF-α can synergistically induce mesenchymal phenotypic changes in TGF-β1 treated IEOs derived from the mouse colon

**Figure 4:**
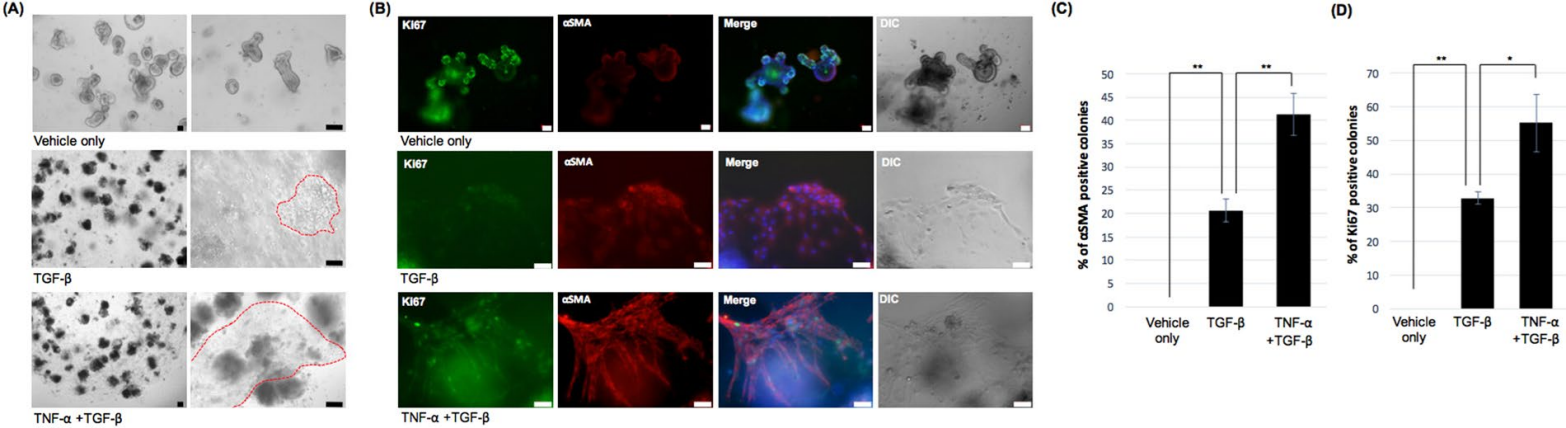
Pretreatment of TNF-α can synergistically induce mesenchymal changes in TGF-β treated IEOs derived from the mouse colon. (**A**) Representative images of mesenchymal changes and migratory cells in cIEOs treated with TNF- α and TGF-β1. Bars, 100 μm. Red arrow, migrating cells. (**B**) The expression of α-SMA and Ki67 was examined in TGF-β and TNF-α treated cIEOs. Bars, 50 μm. (**C**) The percentage of α-SMA positive colonies among all the colonies was determined. (**D**) The percentage of Ki67 positive colonies among all the colonies was determined. Data are presented as the mean ± SEM; n = 4 samples per bar, **p < 0.01, *p < 0.05.

The mesenchymal changes of the TGF-β1 treated IEOs derived from the mouse small intestines were synergistically induced by TNF-α pretreatment; therefore, we next examined whether TNF-α pretreatment promotes mesenchymal changes in TGF-β1 treated IEOs derived from the mouse colon (cIEOs). Accordingly, we did the cIEO experiments the same as the above experiments. We examined whether mesenchymal changes and migration were observed after TNF-α treatment at 1 day in the cIEO culture and after TGF-β1 treatment at 2 days in the cIEO culture. The mesenchymal changes and migratory cells in the TNF-α and TGF-b treated cIEOs were more frequent than in the TGF-β1 treated cIEOs without TNF-α (Fig. 4A and Supplementary Figure 1). When TNF-α and TGF-β1 treated cIEOs were immunostained for α-SMA and Ki67, almost all the mesenchymal changed colonies exhibited mesenchymal and proliferation markers. An approximately a 2.0-fold increase in the a-SMA + colonies was observed in the TNF-α and TGF-β1 treated group relative to the TGF-β1 treated group (n = 4, p < 0.01) (Fig. 4C), and a 1.7-fold increase in the Ki67 positive colonies were observed in the TNF-α and TGF- β treated group relative to the TGF-β1 treated group (n = 4, p < 0.05) (Fig. 4D). Taken together, these data indicate that TNF-α pretreatment was effective in inducing mesenchymal changes in TGF-β1 treated cIEOs similar to the IEOs.

### TNF-α alone induces a spherical morphologic change with a reduction in budding in the IEOs

**Figure 5:**
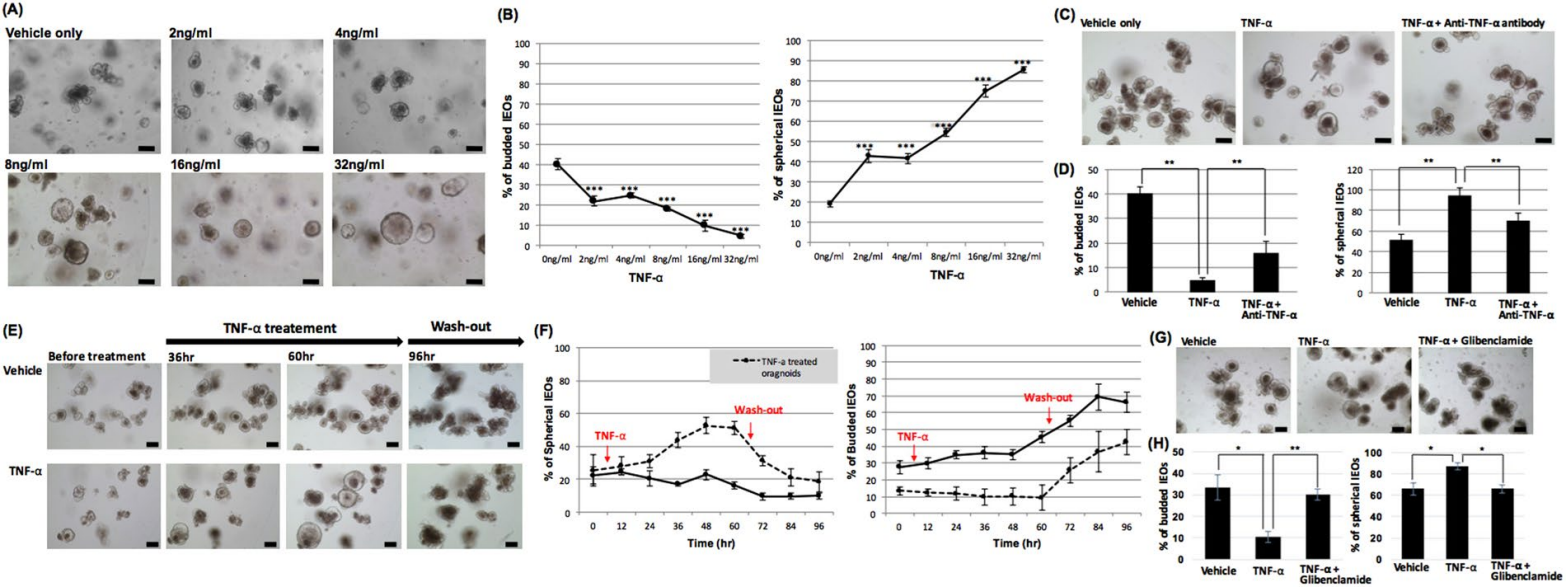
TNF-α treatment results in spherical morphologic changes in IEOs. (**A**) Representative images showing IEOs with TNF-α treatment. Bars, 100 μm (**B**) The percentage of budded (left) or spherical (right) IEOs among all the IEOs that were treated with TNF-α was determined. (**C**) Representative images showing TNF-α treated IEOs with or without TNF-α neutralizing antibodies. Bars, 100 μm (**D**) The percentage of budded (left) or spherical (right) IEOs among all the IEOs that were treated with TNF-α neutralizing antibody was determined. (**E**) Time-course images of when TNF-α was removed from the TNF-α treated IEO media. Bars, 100 μm (**F**) The percentage of spherical (left) or budded (right) IEOs among all the IEOs that were initially treated with TNF-α and then removed was determined. (**G**) Representative images of TNF-α treated IEOs with or without glibenclamide. Bars, 100 μm. (**H**) The percentage of budded (left) or spherical (right) IEOs among all the IEOs treated with glibenclamide was determined. Data are presented as the mean ± SEM; n = 4 samples per bar, ***p < 0.001, **p < 0.01.

Previous studies on the role of TNF-α in intestinal epithelium have suggested that TNF- α could be a mediator of diarrhea during intestinal inflammation because of the opening of ion channels and the loosening of cell-cell junctions^26–28^. However, no information is available regarding the effect of TNF-α on IEOs. To confirm the functionality of TNF-α in IEOs, we examined whether the treatment of TNF-α induces morphological changes in the IEOs. To test this, we treated the IEOs with mouse TNF-α at 1 day after the initial seeding and performed morphological analyses at 2 days after the TNF-α treatment. We observed two morphological characteristics of the TNF-α treated IEOs: irregularly budded shaped IEOs and smoothly spherical shaped IEOs (Fig. 5A). The number of budded IEOs in the high dose TNF-α treated IEOs was less than that of the low dose TNF-α treated IEOs. Conversely, the number of spherical changed IEOs was greater in the high dose TNF-α treated IEOs than in the low dose TNF-α treated IEOs (Fig. 5B). The increase in the number of spherical and budded IEOs by TNF-α was nullified by the treatment with TNF-α neutralizing antibody indicating that TNF-α was responsible for the morphological changes in the TNF-α treated IEOs (Fig. 5C and D).

We speculated that the spherical morphology following the TNF-α treatment may indicate that TNF-α induces the entry of water into the lumen from the outside of the IEOs by opening ion channels and loosening intercellular junctions. If this hypothesis is correct, the TNF-α induced morphology should return to the original shape after removing TNF-α from the IEO culture medium. When TNF-a was removed at 2.5 days after the TNF-α treatment, shrinkage of the spherical IEOs was observed within 12 hours after removing the TNF-α, and almost all the shrinking IEOs were changed into multi-lobed IEOs in 1 day (Fig. 5E and F).

TNF-α stimulates the cystic fibrosis transmembrane conductance regulator (CFTR)- mediated fluid secretion^29^. We hypothesized that the spherical morphology changes of IEO by TNF-α may be a phenomenon that occurs by opening the CFTR. To confirm this hypothesis, glibenclamide^30,31^, a CFTR inhibitor, was treated with TNF-α. IEOs treated with TNF-α and glibenclamide showed the decreased number of spherical IEOs and the increased number of budded IEOs (Fig. 5G and H).

All together, these data indicate that TNF-α treatment of IEOs induces a spherically morphologic change, and although not completely, the IEOs after TNF-α removal immediately revert to their original morphology and start to bud like the vehicle-only treated IEOs. In particular, when IEOs were treated with the CTFR inhibitor, the effects of TNF-α were reduced. Therefore, these results suggest that TNF-α could be an inducer of watery diarrhea by opening ion channels and loosening cell-cell junctions in IEOs similar to previous studies using colon cell lines and animals.

### TNF-α promotes mesenchymal phenotypic changes in TGF-β1 treated IEOs by regulating gene expression associated with EMT

**Figure 6:**
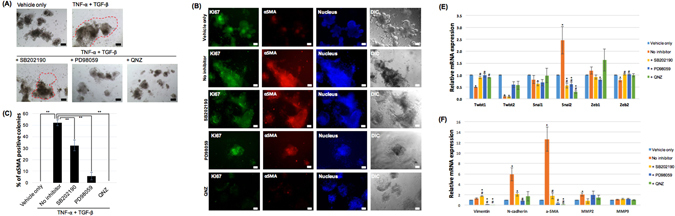
TNF-α promotes mesenchymal phenotypic changes in TGF-β1 treated IEOs by regulating gene expression associated with EMT. (**A**) Representative images of the IEOs following TNF-α, TGF-β treatment with or without inhibitors. Bars, 100 μm. (**B**) The percentage of α-SMA positive colonies was determined. Data were presented as the mean ± SEM; n = 4 samples per bar, **p < 0.01. (**C**) The expression of α-SMA and Ki67 was examined in TGF-β1 treated IEOs with or without inhibitors. Bars, 50 μm. (**D**) Relative mRNA expression for EMT-related transcription factors was quantified after TNF-α treatment in IEOs. (**E**) Relative mRNA expression for EMT effector genes was quantified after TNF-α treatment in IEOs. Data are presented as the mean ± SEM; n = 3 samples per bar, *p < 0.05, control versus TNF-α or inhibitors; ^#^*p* < 0.05, TNF-α versus inhibitors.

We have previously demonstrated that treating TNF-α to the IEOs has a synergistic effect on mesenchymal phenotypic changes of TGF-β1. In order to elucidate the mechanism of synergism by TNF-α, we examined the contribution of TNF-α-activated signaling pathway on mesenchymal phenotypic changes by TGF-β1. The signaling pathway activated by TNF-α is known to be ERK, p38, and NF-kB pathway^32^. To observe the influences of TNF-α activated pathway on EMT formation, PD98059 as an ERK inhibitor, SB202190 as a p38 inhibitor, and QNZ as an NF-kB inhibitor were treated with TNF-α and then treated with TGF-β1. When TNF-α pathway was blocked by SB202190, mesenchymal phenotypic changes were observed but not in PD98059 and QNZ treated groups (Fig. 6A). To quantify EMT efficiency, the IEOs treated with inhibitors were immunostained for α-SMA (Fig. 6B). Approximately, 0.62-fold decrease in the α-SMA+ colonies was observed in the SB202190 treated group while 0.11-fold decrease in PD98059 treated group. Morevoer, no α-SMA+ colony in QNZ treated group relative to the TNF-α and TGF-β1 treated group was observed (n = 4, p < 0.01) (Fig. 6C).

It has been known that TNF-α promotes EMT by up-regulating transcription factors such as Twist1/2, Snai1/2 and Zeb1/2 in renal cell carcinoma, breast cancer cell and colon cancer cell^33–36^. These regulators induce EMT by increasing the expression of effector genes such as N-cadherin, MMPs, vimentin, and α-SMA^37^. To examine the effect of TNF-α on TGF-β1 mediated mesenchymal change, we quantified the expression level of EMT-related transcription factors in IEOs treated with TNF-α. mRNA expression of Twist1/2 and Zeb2 were significantly decreased and Snai2 was increased compared to that of untreated IEO. These expression changes were reversed when the TNF-α-activated pathway inhibitors were treated (Fig. 6D). Next, the expression of EMT effector genes of the IEOs was examined after TNF-α was treated. mRNA expression of N-cadherin, a-SMA, and MMP2 increased significantly, but vimentin and MMP9 was not changed. Similar to the above, expression of TNF-α-mediated effector genes was reversed when the TNF-α-activated pathway inhibitors were treated. (Fig. 6E).

As illustrated here, TNF-α treatement in the IEOs induces up-regulation of transcription regulators like Snai2 and EMT effectors such as N-cadherin, a-SMA and MMP2. However, some EMT-related genes do not alter or decrease expression. These results suggest that TNF-α alone is not sufficient to elicit mesenchymal change in the IEOs, but it may provide a mechanism to synergistically induce EMT when treated with TGF-β1 together.

## Discussion

Recently, Clever’s group and others have established long-term culture conditions, under which isolated intestinal crypts generate IEOs with crypt-villus structures^12,38^. This 3D culture and outgrowth of intestinal crypt stem cells into organoids have offered new possibilities to develop intestinal disease models of primary intestinal epithelial cells with *in vivo* physiology^13,38^. Using this system here, we generated a new IEO model of intestinal fibrosis based on EMT type 2 (OEMT model).

Previous studies have used TGF-β1 as a potent EMT inducer in several epithelial cell lines^6,18,21^. Therefore, we attempted to induce EMT in IEOs by TGF-β1 treatment. However, TGF-β1 mainly disrupted the IEOs in a concentration- and time-dependent manner. Disrupted IEOs appeared in nearly 90% of the colonies after 48 h of TGF-β incubation (1ng/ml). Interestingly, in approximately 10% of the disrupted IEO derived colonies, we found spindle-shaped, mesenchymal like cells migrating. Those colonies were positive for both vimentin and α-SMA, suggesting EMT-derived mesenchymal cells. However, the efficiency of EMT (percentage of α-SMA positive colonies) was not relatively high shown in Fig. 3 (14% in IEO derived colonies) and 4 (21% in cIEO derived colonies). These findings suggest that although TGF-β1 is an essential factor for inducing EMT, it cannot efficiently induce EMT by itself. TGF-β1 can induce EMT in IEOs but not enough for EMT. Previously, the roles of TGF-β1 both in EMT and apoptosis in epithelial cells have been documented^21^. In EMT associated with fibrosis (EMT type 2), numerous molecules (including TGF-β by inflammatory cells) cause degradation of the basement membrane so epithelial cells undergo apoptosis (the majority of cells) or EMT (the minority of cells)^17^. Yang *et al*. demonstrated that TGF-β1 is able to induce not only apoptosis but also EMT in epithelial cells through a cell cycle-dependent mechanism by showing that TGF-β1 induced apoptosis in cells synchronized at the G2/M phase but EMT at the G1/S phase^21^. Thus, the cell cycle phase of epithelial cells in IEOs could be a factor, partly explaining why the same type of IEOs responds differently (apoptosis or EMT) to the same factor (TGF-β1).

Next, we tried to improve the efficiency of EMT in TGF-β1treated IEOs. Based on previous reports that pro-inflammatory cytokines (TNF-α and IL-1) induce EMT for the progression of organ fibrosis through the activation of Snail by NF-kB^24^, we evaluated whether macrophage-released cytokines stimulated by LPS can affect the efficiency of EMT in TGF-β1 treated IEOs using a co-culture system. As expected, co-culture with macrophages improved the EMT efficiency by 58% compared with TGF-β1 treated IEOs without co-culture. Co-culture with macrophages dramatically increased the concentration of TNF-α in the growth media of the IEOs. These data infer that TGF-β1-induced EMT in IEOs is accelerated dramatically by the presence of LPS activated macrophages, and we identified TNF-α as a possible soluble factor, which is produced by macrophages, that accelerates EMT. TNF-α released from macrophages was shown to promote EMT in human colonic organoids derived from colon carcinoma cell lines^25^.

Our results suggest a critical role for TNF-α in stimulating EMT in IEOs. By sequential treatment of TNF-α and TGF-β1, we improved the efficiency of EMT by 64% in IEOs and by 95% in cIEOs compared to the TGF-β1 treatment alone. Finally, EMT appeared in 23% of the IEOs and in 41% of the cIEOs in our OEMT model. Then, what is the mechanism by which TNF-α augments the EMT in TGF-β1 treated IEOs? Based on the previous studies showing that TNF-α confers positive effects toward EMT by inducing various genes, we speculated that the increased EMT efficiency due to TNF- α treatment in the IEOs may be linked to the induction of EMT-associated genes. It was supported by the following results: IEOs treated with TNF-α increased the expression of EMT-associated genes such as snai2, N-cadherin, a-SMA and MMP2. This result also suggests the mechanism by which TNF-α treatment increase efficiency of TGF-β1 mediated EMT in IEOs. Recent studies have suggested that the p38 MAPK pathway is required for TGF-β-mediated EMT^39,40^. Treatment of organoids with TNF-α alone elevated the p38 activity but was not sufficient to induce EMT^25^. However, the elevated p38 activity observed in the presence of both TNF-α and TGF-β1 augmented EMT because this effect was blocked by the p38 inhibitor^25^. This report suggests that synergistic p38 activation could be a mechanism by which TNF-α augments EMT in TGF-β1 treated IEOs. We also confirmed that pathway block by the p38 inhibitor resulted in a reduction of EMT efficiency and a reversal of the EMT-associated genes induced by TNF-α. In other studies, ERK and NF-kB pathway in addition to p38 pathway has also been associated with regulation of EMT-associated genes by TNF-α^35,36^. Indeed, we detected that blockage of the TNF-α mediated pathway by NF-kB and ERK inhibitor reduces the expression of EMT-related gene by TNF-α and reduced efficiency of EMT in IEOs. In conclusion, the activation of various pathways by TNF-α promotes the expression of EMT-associated genes, which may help to improve the efficiency of EMT by TGF-β. Further studies are needed to determine which genes are crucial to increasing EMT efficiency by TGF-β.

Another possible mechanism is that TNF-α induces the dissolution of cell-cell junctions in epithelial cells, which in turn could accelerate the TGF-β-mediated EMT. The loss of apical-basolateral polarity and cell-cell contacts are one of the key mechanisms initiating EMT^41^. One of the early events in EMT is the disassembly and disruption of tight junction (TJ) proteins such as zonula occludens (ZO)-1 and claudins^41^. Several studies have shown that TNF-α causes a disruption of the intestinal epithelial tight junction (TJ) barriers^42–44^. TNF-α induced NF-kB activation was required for the down regulation of ZO-1 proteins and for the alteration in junctional localization of the ZO-1 proteins^42^. Although the expression pattern of TJ proteins in TNF-α treated IEOs was not evaluated in this study, we identified that TNF-α alone induced a spherical morphologic change in the IEOs shown in Fig. 5. We speculate that this spherical morphologic change could occur with intraluminal fluid expansion by an influx of fluid from the outside media of the organoids through an increased TJ permeability. In the process, TNF-α induced disruption of intestinal epithelial TJ barriers could contribute to increasing the TJ permeability. Not only modulating the effect of epithelial permeability but also the secretory effect of TNF-α may contribute to the spherical morphologic change in the IEOs. TNF-α is known to cause the secretion of chloride and water from intestinal epithelium, leading to diarrhea, in the human distal colon^45,46^. In summary, the TNF-α induced combined effect of increased permeability and secretion of chloride and water from epithelial cells may lead to the spherical morphologic change in the IEOs.

Despite great advancements in generating IEO culture models recapitulating human *in vivo* physiology, an appropriate organoid-based intestinal fibrosis model is still not available. A recent study developed an organoid-based intestinal fibrosis model derived from pluripotent stem cells^4^. However, presently, generating a pluripotent stem cell-derived organoid model is not available in many laboratories due to the high costs and long duration as well as the advanced technology required^14,15^. The OEMT model described in this study can be easily generated with a simple method using the sequential treatment of TNF-α and TGF-β1 on IEOs derived from intestinal crypts. The possibility to culture this OEMT model in 48-well plates, and possibly even smaller formats, can enable the screening of a large number of anti-fibrotic drug candidates inhibiting EMT associated fibrosis to generate quantitative data with an economical method.

In conclusion, this study describes for the first time the use of *ex vivo* cultured IEOs to investigate EMT associated with intestinal fibrosis. This model may represent not only a cost-efficient alternative method to *in vivo* studying of drugs with anti-fibrotic activity in intestinal fibrosis animal models but also may be a bridge model to develop a human organoid-based intestinal fibrosis model.

## Materials and Methods

### Animals

This study was approved and conducted according to regulations and guidelines of the CHA University Institutional Animal Care and Use Committee. All efforts were made to minimize animal suffering and to reduce the number of animals used. Male C57Bl/6 mice were obtained from the Orient Bio (Seongnam, Korea) and maintained in the animal facility of CHA University under 12-h light/dark cycle with food and water available ad libitum.

### Preparation of IEOs derived from mouse small intestines

Preparation of IEOs was done with 5–7 week-old C57Bl/6 mice weighing 20~25 g each. Mouse small intestines were removed after sacrifice by cervical dislocation. Small intestines were cut longitudinally from the proximal end to distal end and cut transversely into approximately 5 mm long pieces. The small intestine pieces were washed with ice-cold Dulbecco’s Phosphate-Buffered Saline (DPBS) several times until their supernatant became clear enough. Crypts were then isolated by treatment with Gentle Cell Dissociation Reagent (StemCell Technologies, Cambridge, MA) and filtering with a 70 um cell strainer. Isolated crypts were mixed with Matrigel (BD Biosciences, Franklin Lakes, NJ) at a ratio of 1:1 and plated in 48-well plates. After polymerization of the Matrigel by incubating at 37 °C for 10 minutes, 400 ul of Intesticult™ OGM Mouse Basal Medium (StemCell Technologies, Cambridge, MA) were added as culture medium, and then, the plates were maintained in a humidified incubator (5% CO_2_ in air) at 37 °C. The culture medium was replaced every 2 days.

### Preparation of IEOs derived from the mouse colon

Except for the isolation of the crypts, preparation of the IEOs from the mouse colon was performed as described above. For the isolation of the crypts from the colon, colon pieces were incubated with ice-cold crypt chelating buffer (2 mM EDTA in DPBS) for 30 minutes and vigorously inverted for several minutes with dissociation buffer (2 g of D-Sorbitol and 3 g of Sucrose in 200 ml of DPBS). Isolated crypts were mixed with Matrigel (BD Biosciences, Franklin Lakes, NJ), plated in 48-well plates and maintained in Intesticult™ OGM Mouse Basal Medium.

### Production of the OEMT by sequential treatment with cytokines and Inhibition assay

Mouse TGF-β1 (R&D Systems, Minneapolis, MN) and Recombinant Mouse TNF-α (Biolegend, San Diego, CA) were used in this experiment. IEOs were seeded the day before in IEO culture medium containing 32 ng/ml TNF-α. Two days after the TNF-α treatment, the IEOs were simultaneously treated with 8 ng/ml TGF-β1 and 32 ng/ml TNF- α in IEO culture medium. For inhibition assays, the inhibitors were treated with 32 ng/ml TNF-α in IEOs. The concentrations of inhibitors used for inhibitor assays were as follows; 10 µM SB202190 (Sigma-Aldrich, St. Louis, MO), 20 µM PD98059 (Sigma-Aldrich), 10 µM QNZ (Sigma-Aldrich), 10 µM Glibenclamide (Sigma-Aldrich).

### Production of OEMT by IEOs co-cultured with RAW264.7 cells

The murine macrophage cell line RAW264.7 (ATCC, Rockville, MD) was cultured in a humidified incubator (5% CO_2_ in air) at 37 °C. 1 × 10^4^ RAW264.7 cells were seeded in a 0.4 um-pore-size Transwell. The next day, RAW264.7 cells were treated with 2 µg/ml Lipopolysaccharide (LPS) from Escherichia coli O111:B4 (Sigma-Aldrich) to induce pro-inflammatory cytokines. For OEMT induction, the transwell with the RAW264.7 cells were transferred to the wells with the IEOs treated with 8 ng/ml of recombinant Mouse TGF-β1.

### Immunofluorescence

Culture medium was removed, and the organoids in Matrigel were washed with DPBS. Fixation was done with 4% Paraformaldehyde until the Matrigel was disintegrated. Permeabilization was performed with 0.1% Tween-20 and 0.2% Triton-X100 in PBS buffer, and then, 5% BSA in DPBS was used to reduce non-specific binding. Samples were covered with primary antibodies at 4 °C overnight. The next day, the samples were embedded with secondary antibodies at room temperature for 2 h. Antibodies used for the staining were as follows: Mucin 2 (Santa Cruz Biotechnology, Santa Cruz, CA), Ki67 antibody (Abcam, Cambridge, MA), Lgr5 (Abgent, San Diego, CA), E-cadherin (Santa Cruz Biotechnology), ChromograninA (Santa Cruz), β –catenin (Abcam), Lysozyme (Diagnostic Biosystems, Fremont, CA), α-SMA (Sigma-Aldrich), Vimentin (BD Biosciences), and Alexa Flour 488 conjugated anti-rabbit IgG and Alexa Flour 594 conjugated anti-mouse IgG (Life Technologies, Gaithersburg, MD).

### Enzyme-linked immunosorbent assay (ELISA)

The IEO growth medium from the IEOs co-cultured with the RAW264.7 cells was collected at 1 day after the LPS treatment. The amount of TNF-α protein was measured with ELISA (BioLegend) following the manufacturer’s instructions.

### Quantitative reverse transcriptase-polymerase chain reaction (qRT-PCR) analysis

The IEOs were treated Cell Recovery Solution (Corning Incorporated, Corning, NY) to remove Matrigel, and total RNA was extracted by using the MagListo™ 5 M Cell Total RNA Extraction Kit (Bioneer, Daejeon, Korea). The total RNA extraction procedure was followed by manufacturer’s guideline. RNA was synthesized to cDNA by using AccuPower® RocketScript™ Cycle RT Premix (Bioneer). Quantitative polymerase chain reaction was performed by mixing cDNA with AccuPower® 2X Greenstar qPCR Master Mix (Bioneer). The mRNA expression level was measured by Rotor Gene Q (Qiagen, Hilden, Germany). Data was exported from Rotor Gene Q Series Software (Qiagen). The primer pairs used for qRT-PCR were as follows: a primer pair for mouse Twist1, forward 5′-AGCGGGTCATGGCTAACG-3′, reverse 5′-GGACCTGGTACAGGAAGTCGA-3′; a primer pair for mouse Twist2, forward 5′-CGCTACAGCAAGAAATCGAGC-3′, reverse 5′-GCTGAGCTTGTCAGAGGGG-3′; a primer pair for mouse Snai1, forward 5′-TCTGAAGATGCACATCCGAAGCCA-3′, reverse 5′-AGGAGAATGGCTTCTCACCAGTGT-3′; a primer pair for mouse Snai2, forward 5′-AGATGCACATTCGAACCCAC-3′, reverse 5′-GTCTGCAGATGAGCCCTCAG-3′; a primer pair for mouse Zeb1, forward 5′-CGGTGCCAAGAACTGCTGGCA-3′, reverse 5′-CGGCGGTGTCTTGTTGCTGC-3′; a primer pair for mouse Zeb2, forward 5′-ACGTCAGTCCGTCCCCAGGTT-3′, reverse 5′-GAGTGTCTGGAGGCAGGACCGT-3′; a primer pair for mouse α-SMA, forward 5′-GCATCCACGAAACCACCTA-3′, reverse 5′-CACGAGTAACAAATCAAAGC-3′; a primer pair for mouse MMP2, forward 5′-CAACTACGATGATGACCGCAA-3′, reverse 5′-GTGTAAATGGGTGCCATCAGG-3′; a primer pair for mouse MMP9, forward 5′-AGTTGTGGTCGCTGGGCAAA-3′, reverse 5′-CTACACCAAGGCGTGCCGTC-3′; a primer pair for mouse vimentin, forward 5′-CCCTGAACCTGAGAGAAACTAAC-3′, reverse 5′-GGTCATCGTGATGCTGAGAAG-3′; a primer pair for mouse N-cadherin, forward 5′-AGAAGGTGGAGGAGAAGAAGAC-3′, reverse 5′-TGTGGCTCAGCATGGATAGG-3′; a primer pair for mouse GAPDH, forward 5′-AACTTTGGCATTGTGGAAGG-3′, reverse 5′-ACACATTGGGGGTAGGAACA-3′.

### Statistical Analysis

Differences between two groups were assessed by the Student’s t-test. Three or more groups were compared with one-way ANOVA followed by the Student-Newman-Keuls post hoc test. Differences were considered statistically significant at *p* < 0.05.

## Electronic supplementary material

Supplementary information

Supplementary Movie 1. Treatment of TNF-α and TGF-β can induce mesenchymal changes in cIEOs
