# Author Correction: Organoid-based epithelial to mesenchymal transition (OEMT) model: from an intestinal fibrosis perspective

**DOI:** 10.1038/s41598-018-22624-y

**Published:** 2018-03-06

**Authors:** Soojung Hahn, Myeong-Ok Nam, Jung Hyun Noh, Dong Hyeon Lee, Hyun Wook Han, Duk Hwan Kim, Ki Baik Hahm, Sung Pyo Hong, Jun-Hwan Yoo, Jongman Yoo

**Affiliations:** 1 Department of Microbiology, School of Medicine, CHA University, Seongnam, South Korea; 2 Institute of Basic Medical Sciences, School of Medicine, CHA University, Seongnam, South Korea; 3 Department of Physiology, School of Medicine, CHA University, Seongnam, South Korea; 4 Department of Preventive Medicine, School of Medicine, CHA University, Seongnam, South Korea; 5 Department of Gastroenterology, CHA Bundang Medical Center, CHA University, Seongnam, South Korea

Correction to: *Scientific Reports* 10.1038/s41598-017-02190-5, published online 26 May 2017

This Article contains typographical errors in the Results section.

“No mesenchymal changes and migratory cells was observed when TGF-β1 was treated first and then treated with TNF-α. The similar results were shown in immunostaining with α-SMA and also in quantification of α-SMA positive colonies.”

should read:

“No mesenchymal changes and migratory cells was observed when TGF-β1 was treated first and then treated with TNF-α (Fig. 3E). The similar results were shown in immunostaining with α-SMA (Fig. 3F)and also in quantification of α-SMA positive colonies (Fig. 3G).”

“When TNF-α and TGF-β1 treated cIEOs were immunostained for α-SMA and Ki67, almost all the mesenchymal changed colonies exhibited mesenchymal and proliferation markers.”

should read:

“When TNF-α and TGF-β1 treated cIEOs were immunostained for α-SMA and Ki67, almost all the mesenchymal changed colonies exhibited mesenchymal and proliferation markers (Fig. 4B).”

Additionally, this Article contains errors in Figure 6, where the panels are incorrectly labeled. As a result, in the Figure legend,

“(**B**) The percentage of α-SMA positive colonies was determined. Data were presented as the mean ± SEM; n = 4 samples per bar, **p < 0.01. (**C**) The expression of α-SMA and Ki67 was examined in TGF-β1 treated IEOs with or without inhibitors. Bars, 50 μm.”

should read:

“(**B**) The expression of α-SMA and Ki67 was examined in TGF-β1 treated IEOs with or without inhibitors. Bars, 50 μm. (**C**) The percentage of α-SMA positive colonies was determined. Data were presented as the mean ± SEM; n = 4 samples per bar, **p < 0.01.”

The correct Figure 6 appears below as Figure 1.

**Figure 1:**
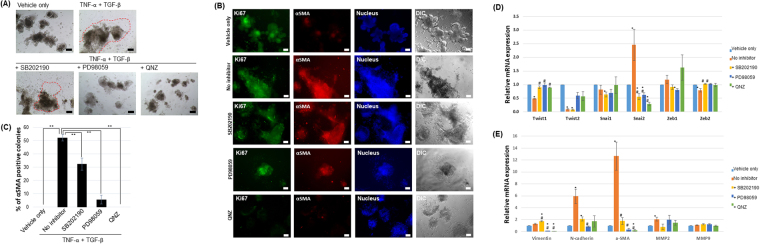
TNF-α promotes mesenchymal phenotypic changes in TGF-β1 treated IEOs by regulating gene expression associated with EMT. (**A**) Representative images of the IEOs following TNF-α, TGF-β treatment with or without inhibitors. Bars, 100 μm. (**B**) The expression of α-SMA and Ki67 was examined in TGF-β1 treated IEOs with or without inhibitors. Bars, 50 μm. (**C**) The percentage of α-SMA positive colonies was determined. Data were presented as the mean ± SEM; n = 4 samples per bar, **p < 0.01. (**D**) Relative mRNA expression for EMT-related transcription factors was quantified after TNF-α treatment in IEOs. (**E**) Relative mRNA expression for EMT effector genes was quantified after TNF-α treatment in IEOs. Data are presented as the mean ± SEM; n = 3 samples per bar, *p < 0.05, control versus TNF-α or inhibitors; ^#^*p* < 0.05, TNF-α versus inhibitors.

