# Supplementary material for: Organoid-based epithelial to mesenchymal transition (OEMT) model: from an intestinal fibrosis perspective
Source: Sci Rep. 2017 May 26;7:2435. doi: 10.1038/s41598-017-02190-5 (PMC5446415; doi:10.1038/s41598-017-02190-5)
Supplement: Supplementary file 1 — Supplementary information [file 41598_2017_2190_MOESM1_ESM.doc]

**Supplementary information**

**Organoid-based epithelial to mesenchymal transition (OEMT) model: from an intestinal fibrosis perspective**

Soojung Hahn, Myeong-Ok Nam, Jung Hyun Noh, Dong Hyeon Lee, Hyun Wook Han, Duk Hwan Kim, Ki Baik Hahm, Sung Pyo Hong, Jun-Hwan Yoo, Jongman Yoo

**Supplementary Movie 1. Treatment of TNF-α and TGF-β can induce mesenchymal changes in cIEOs.**

The movie shows transmitted light time-lapse images of cIEOs after treatment of TNF-α and TGF-β. The time-lapse covers a period of 48 hours, with approximately 3 hours elapsed time per second of movie. It can be observed that mesenchymal change is induced when the TNF-α and TGF-β are treated with IEO.
